# Extra-uterine twin pregnancy: case report of spontaneous bilateral tubal ectopic pregnancy

**DOI:** 10.11604/pamj.2015.20.435.6338

**Published:** 2015-04-30

**Authors:** Ben Haj Hassine Amine, Siala Haythem

**Affiliations:** 1 Department of Gynecology and Obstetrics, Principal Military Hospital of Instruction of Tunis, Tunis, Tunisia

**Keywords:** Ectopic, twin, tubal pregnancy

## Abstract

Spontaneous bilateral ectopic pregnancy is the rarest form of ectopic pregnancy. Bilateral tubal pregnancies in the absence of preceding induction of ovulation are a rare occurrence, with an estimated incidence of 1 in 725 to 1 in 1580 ectopicpregnancies. They are usually diagnosed at the time of surgery. We report a case of spontaneous bilateral tubal pregnancies diagnosed intraoperatively. A 33-year-old patient was admitted after light vaginal bleeding, 35 days after her last menstruation. A bilateral salpingectomy was performed without complication and the pathology report confirmed the diagnosis. This is an unusual case of early diagnosis. The diagnosis of bilateral tubal pregnancy is usually madeintraoperatively. This underscores the importance ofidentifying and closely examining both tubes at the time ofsurgery, even in the presence of significant adhesive disease.

## Introduction

Bilateral tubal pregnancies in the absence of preceding induction of ovulation are an extremely unusual occurrence. The incidence of simultaneous bilateral tubal pregnancies has been reported to range from 1 per 725 to 1per 1580 ectopic pregnancies [1]. This is thought to correspond to an occurrence of one per every 200 000 live births [2]. Authors report an unusual case of ectopic pregnancy in which patient had spontaneous bilateral tubal ectopic pregnancy which presented with right tubal rupture and subsequently emergency exploratory laparotomy revealed bilateral tubal mass, which on histopathological examination confirmed tubal pregnancy.

## Patient and observation

A 33-year-old gravida 8, para 3 woman was referred to the gynecology service with an approximate gestational age of 9 weeks and 2 days. Presenting complaints included vaginal bleeding and intermittent lower abdominal cramping of 7 days duration; fever 38^°^c The patient was hemodynamically stable. The initial level of serum hCG was positive. On pelvic examination, there was mild spotting, the cervical os was closed and the cervix tender on transverse motion. The uterus was bulky; there was fullness in all the fornices with tenderness; and the adnexae were difficult to palpate. Haematological examination showed: white cell count 22 × 10^3^cells/L, haemoglobin 8.5 g/dL and haematocrit 29,4%. A transvaginal pelvic ultrasound examination revealed an empty uterus with a right adnexal mass with sac gestationnel, positive cardiac activity, LCC 14mm and an important amount of fluid collection was present in the pouch of Douglas. The patient's past gynaecologic history included a previous therapeutic abortion. She had no history of sexually transmitted infections or previous abdomino-pelvic surgery. She was otherwise healthy.

**Figure 1:**
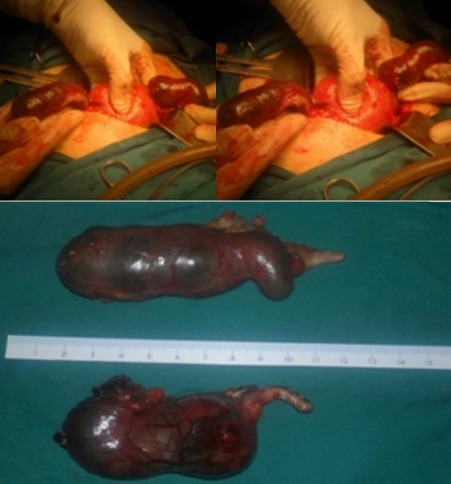
Bilateral tubal pregnancy

The patient was counselled concerning the possibility of anectopic pregnancy, and informed consent for laparotomy exploration with the possible need for salpingostomy orsalpingectomy was obtained. Emergency exploratory laparotomy revealed haemoperitoneum of approximately 2000 mL. There was a ruptured fimbrial ectopic pregnancy with active bleeding on the right side. The left tube showed an intact ectopic pregnancy 10 cm in the ampullary region that was bleeding and forming an organized haematoma at the fimbrial end (Figure 1). We considered it possible that the patient had bilateral spontaneous ectopic pregnancies and In view of these findings, we decided to perform bilateral retrograde salpingectomy. The patient received 2 units of type O Rh negative blood. Postoperative follow-up was uneventful and the patient was discharged on the 5^th^ day post-operation. The pathology report confirmed the diagnosis of spontaneous bilateral tubal pregnancies, showing blood clot admixed with chorionic villi in the tissue removed from the right tube. The tissue obtained from the left tube showed multiple fragments of fetal tissue, including the vertebral column, neurological structures, liver, intestine, umbilical cord, and chorionic villi.

## Discussion

There are three possible explanations for a bilateral ectopic pregnancy: 1) simultaneous multiple ovulation, 2) sequential impregnation or 3) transperitoneal migration of trophoblastic cells from one extrauterine pregnancy to the other tube with implantation there [3]. Spontaneous bilateral ectopic pregnancy is rare, therefore preoperative diagnosis is uncommon indicating limitations of ultrasonography. In our case also preoperative ultrasound failed to demonstrate bilateral tubal ectopic pregnancy. The most common method of diagnosing the second ectopic is direct inspection of contralateral tube in the operating room or laparoscopic examination. The frequency of bilateral ectopic pregnancy has been estimated at 1/200 000 uterine pregnancies and 1/725-1/1580 ectopic pregnancies. In the past 20 years a 3-fold increase in the incidence has been observed [4]. Heterotopic as well as bilateral tubal ectopic pregnancies are seen after the introduction of assisted reproductive treatment [5, 6]. The occurrence of spontaneous bilateral ectopic pregnancy is, however, exceedingly rare [4, 7]. Comprehensive clinical guidelines for the treatment of ectopic pregnancy have been published by the Royal College of Obstetricians and Gynaecologists [8]. Because of its rarity, synchronous ectopic pregnancy is not covered, but the principles of treatment can still be applied. Laparoscopic surgical treatment is preferred to open procedures, because the patient recovers more quickly and subsequent rates of intrauterine and ectopic pregnancy are similar. Our patient, because of her acute symptoms, was not suitable for either laparoscopic surgery or medical management with methotrexate. This case demonstrates the importance of thoroughly examining the entire pelvis at the time of exploratory laparotomy undertaken for a suspected ectopic pregnancy. The necessity of carefully examining both the adnexae, as postulated by Sherman cannot be overemphasized [9]. The patient who has had one ectopic pregnancy is at risk of having another in future and also at the same time.

## Conclusion

Ectopic pregnancy is still an important cause of maternal mortality. The diagnosis of bilateral tubal pregnancy is usually made intraoperatively. This underscores the importance of identifying and closely examining both tubes at the time of surgery, even in the presence of significant adhesive disease.
